# Effect of *Calotropis procera *latex extracts on the hypothalamic TNFα and PGE_2 _levels in the rat model of yeast-induced pyrexia

**DOI:** 10.1186/cc11732

**Published:** 2012-11-14

**Authors:** B Guruprasad, P Chaudhary, VL Kumar

**Affiliations:** 1 All India Institute of Medical Sciences, New Delhi, India

## Background

Sepsis, a common cause of morbidity and mortality in critically ill patients, is associated with systemic inflammatory response syndrome due to upregulation of cyclooxygenase-2 and increase in the levels of PGE_2_. It is also associated with increase in the levels of proinflammatory cytokines like TNFα and IL-1β. *Calotropis procera *is a plant that grows in the wild producing latex. The aqueous and methanolic extracts of dried latex of this plant (AqDL and MeDL) and proteins isolated from the fresh latex (LP) have shown anti-inflammatory and anti-arthritic properties. AqDL and MeDL are orally effective, LP is effective parenterally. The current study was designed to evaluate the efficacy of these extracts against yeast-induced pyrexia and the levels of TNFα and PGE_2 _in the hypothalamus of rats.

## Methods

Pyrexia was induced in rats by subcutaneous injection of yeast in the nape of the neck and the rectal temperature was measured at 0 hours (basal temperature), 3 hours and 6 hours. Rats were divided into groups (*n *= 6) and were treated with AqDL and MeDL given orally and LP given intravenously at 6 hours. Group I: NC (normal control); Group II: YC (yeast control); Group III: AqDL (200 mg/kg); Group IV: AqDL (400 mg/kg); Group V: MeDL (100 mg/kg); Group VI: MeDL (250 mg/kg); Group VII: LP (5 mg/kg); Group VIII: LP (25 mg/kg); Group IX: paracetamol (PCM 100 mg/kg). Rectal temperature was measured hourly until 9 hours. The levels of TNFα and PGE_2 _were measured in the excised hypothalamus region of the brain using ELISA kits.

## Results

Subcutaneous injection of yeast produced a marked increase in rectal temperature of rats with a maximum effect at 6 hours (101.17°C). Like paracetamol, treatment of rats with AqDL and MeDL produced a significant decrease in body temperature from 101.17°C at 6 hours to 97.9°C and 98.2°C at 9 hours respectively at higher doses and their effect was dose dependent while LP was found to be ineffective. The present study shows that treatment with yeast increased the tissue levels of TNFα (23.78 pg/mg) and PGE_2 _(66.48 pg/mg) as compared with the NC group (16.31 and 41.35 respectively). All of the fractions lowered the hypothalamic TNFα level while a marked reduction in PGE_2 _levels was observed with orally effective fractions, namely AqDL and MeDL.

## Conclusion

Our results demonstrate that the orally administered fractions of latex of *C. procera *are effective in attenuating yeast-induced pyrexia and this effect is mediated through reduction in the levels of PGE_2_.

